# Editorial: New Approaches to Radiation-Therapeutic Agent Cancer Care for Women

**DOI:** 10.3389/fonc.2017.00276

**Published:** 2017-11-16

**Authors:** Charles A. Kunos, Elise C. Kohn

**Affiliations:** ^1^Cancer Therapy Evaluation Program, National Cancer Institute, Bethesda, MD, United States

**Keywords:** rare orphan subsets, women’s cancers, triapine, cervical cancer, ADXS-human papillomavirus, radiochemotherapy clinical trial

**Editorial on the Research Topic**

**New Approaches to Radiation-Therapeutic Agent Cancer Care for Women**

It is estimated that 852,000 women will be diagnosed with cancer in the United States (US), and one-third will die each year (1). Advances in cancer treatment have raised the rate of 5-year cancer survival over the past five decades, and yet, cancer remains the second leading cause of death in American women, resulting in more deaths than the next five causes combined (2). Hence, there is an urgency to develop and execute clinical trials of innovative treatments that more rapidly advance standards of care (3).

We have observed, as we advance our understanding of women’s cancers, substantial differences within cancers to identify either new types or new subtypes of cancer that drive different treatment directions. We hypothesize that more frequently, these rare subsets of women’s cancers will fit definitions of orphan cancer subsets. Such orphan subsets are defined by the US Food and Drug Administration as having fewer than 200,000 affected American women alive at any given time, with *any history* of that unique (women’s cancer) subset over any given year (4). This becomes important as it relates to advancing and bringing to approval treatment opportunities that may be selective to those unique women’s cancers subsets.

Take uterine cervix cancer as an example. In 2017, this women’s cancer takes much less of a toll on American women, where it is only the third most common women’s cancer (12%, 12,820 of 107,470 new women’s cancers) and the fourteenth leading cause of cancer-related death (1.5%, 4,210 of 282,500 any type cancer-related deaths) (1). It was estimated in January 2017 that the number of American women alive who had *any history* of an invasive cervix cancer was about 231,040 (0.1% of 165 million women) (5). Such a complete prevalence rises above the rare orphan subset designation threshold of 200,000 persons. However, this does not incorporate consideration of differentially treated subsets that may fall within the orphan designation threshold.

The US National Cancer Institute’s Cancer Therapy Evaluation Program has supported the development of triapine (3-aminopyridine-2-carboxaldehyde thiosemicarbazone), an inhibitor of ribonucleotide reductase (RNR). RNR is an enzyme overexpressed in uterine cervix cancer that, when inhibited, blocks production of deoxyribonucleotides required for DNA replication or repair, arrests cancer cells at their G1/S cell cycle phase restriction checkpoint, raises the number of unrepaired damaged DNA foci, and promotes cell lethal events (6). Although triapine monotherapy and triapine–cisplatin doublets were ineffective clinically (7, 8), the translation of preclinical success with a triapine–cisplatin–radiotherapy combination was effective yielding a 92% cancer-specific survival in women with newly diagnosed regionally advanced, stage II–IVA, uterine cervix cancers [Kunos and Sherertz; (9)]. This suggests marked potential when compared with 57% survival obtained with cisplatin–radiotherapy treatment among same stage historical cohorts (1). An ongoing randomized clinical trial now tests the efficacy question directly (NCT02466971). This is an example of a focused intervention for a uniquely targeted subset of uterine cervix cancer.

Another example is the development of ADXS-human papillomavirus (HPV) (ADXA11-001), a recombinant live-attenuated *Listeria monocytogenes* that secretes the antigen HPV E7 protein fused to a non-hemolytic virulence factor listeriolysin O (*LM-*LLO-E7). The putative therapeutic effect of ADXS-HPV is its ability to induce E7-specific tumor-infiltrating T-cells, reduce regulatory CD4+ CD25+ T-cells and block tumor blood vessel formation (10). ADXS-HPV has an intended treatment orphan subset of women with stage II–IVA uterine cervix cancers, and an ongoing randomized clinical trial now tests the efficacy of *LM-*LLO-E7 following standard radiochemotherapy (NCT02853604).

Newly diagnosed regionally advanced uterine cervix cancer affects about 4,615 women each year (36%, of 12,820 new cases) or approximately 2.8 per 100,000 women in the US (1). Such a statistic might be better grasped by the following example—if a large football stadium holds 100,000 fans, one would need to fill 1 entire football stadium with female fans to find 3 new cases of regionally advanced uterine cervix cancer. An estimate for the complete prevalence of women alive who have had any history of regionally advanced uterine cervix cancer might be calculated to be 83,174 women (36% of 231,040 women). This fits better under a rare orphan subset guidance (4). Indeed, the 4,615 annual cases make up only 2% of the 231,040 American women alive at any one time with any stage invasive disease. For an intended treatment indication of regionally advanced disease where only 15 triapine infusions are ever given in a lifetime, there remains no reasonable expectation that the costs of research and trials with the drug can be recovered by sales of the drug in the US. This latter notion too fits the rare orphan subset guidance (4).

Finally, why pursue active oncology product clinical development in a rare orphan subset of women’s cancer through clinical trials beyond perceived financial toxicity? There are two reasons: (a) women gain access to newly discovered drugs matched to the underlying biology of their disease much earlier than they otherwise would (if ever), and (b) quality, safety, and efficacy of such drugs can be addressed before widespread use. We advocate that US National Cancer Institute’s Cancer Therapy Evaluation Program programmatic collaborations leverage bench science and trials to foster better and faster oncology product development in rare orphan subsets. The example of triapine in a rare orphan subset of uterine cervix cancer highlights a potential regulatory pathway where scientific and financial risks are high, but rewards measured in potential efficacy are higher.

## Author Contributions

Conception: CK, EK; writing: CK, EK; review and approval: CK, EK.

## Conflict of Interest Statement

The authors declare that the research was conducted in the absence of any commercial or financial relationships that could be construed as a potential conflict of interest.
